# Tracheal Obstruction by Thyroid Gland Extension into the Trachea after Blunt Tracheal Transection

**DOI:** 10.70352/scrj.cr.24-0072

**Published:** 2025-02-27

**Authors:** Hironori Ishibashi, Michi Aoki, Shunichi Baba, Akihiro Fujita, Kenichi Okubo

**Affiliations:** 1 Department of Thoracic Surgery, Institute of Science Tokyo, Tokyo, Japan; 2 Department of Trauma and Acute Critical Care Center, Institute of Science Tokyo, Tokyo, Japan

**Keywords:** blunt tracheal injury, thyroid gland, venovenous extracorporeal membrane oxygenation

## Abstract

**INTRODUCTION:**

Tracheal injuries due to blunt force trauma are rare yet life-threatening conditions, comprising only 4% of chest trauma cases. Diagnosis is often delayed, increasing the risk of severe complications. This report describes a unique case of tracheal obstruction caused by thyroid gland extension into the trachea following blunt trauma, which was managed successfully with venovenous extracorporeal membrane oxygenation (ECMO) and surgery.

**CASE PRESENTATION:**

A 50-year-old male presented with severe respiratory distress following a seizure-induced fall at his residence. On arrival at the hospital, the patient was in respiratory failure with an SpO_2_ of 92% on a 10 L/min reservoir mask, had severe subcutaneous emphysema, and an upper airway stridor. Computed tomography revealed mediastinal emphysema and a 13-mm endotracheal mass obstructing the trachea. Flexible bronchoscopy indicated a suspected tracheal tumor, but intubation was unsuccessful due to bleeding and obstruction. Emergency tracheostomy was considered but deemed risky because imaging showed that the distal trachea was located near the sternum’s suprasternal margin. The patient’s respiratory distress worsened, and his SpO_2_ dropped to 86%. Venovenous ECMO was then administered, stabilizing his condition. Surgical intervention was performed to address the endotracheal mass and tracheal injury. A transverse neck incision allowed dissection and identification of the tracheal injury, revealing the inferior thyroid gland which extended into the tracheal lumen. Pathological examination confirmed the endotracheal mass as normal thyroid tissue. Tracheal anastomosis was successfully completed, and the patient was discharged on postoperative day 10 without complications.

**CONCLUSION:**

This case highlights an unusual presentation of tracheal obstruction caused by thyroid gland extension into the trachea following blunt trauma. Rapid initiation of ECMO enabled successful airway management and surgical repair. Recognizing atypical presentations of tracheal injuries is critical in trauma cases, as prompt intervention can prevent further complications and improve patient outcomes. This case underscores the importance of tailored airway management and the potential role of ECMO in cases of similar complex airway obstructions.

## Abbreviation

ECMO
: extracorporeal membrane oxygenation

## INTRODUCTION

Blunt tracheal injuries are uncommon but life-threatening, accounting for only 4% of all chest trauma cases, with a significant proportion of patients not surviving hospital admission due to a compromised airway and rapid deterioration.^1)^ Tracheal obstruction due to trauma generally results from tracheal cartilage collapse or hemorrhage. Thyroid gland extension into the tracheal lumen as a result of blunt trauma is rare. Management of airway patency in tracheal injuries is a priority during trauma assessments, as traditional intubation attempts can exacerbate the injury, potentially leading to complete airway disruption. Venovenous extracorporeal membrane oxygenation (ECMO) has emerged as a valuable adjunctive therapy, particularly in cases where securing the airway is complicated by anatomical challenges or risk of further injury, as it allows for temporary respiratory support while stabilizing the patient for surgical intervention. This report presents a unique case of tracheal obstruction caused by thyroid gland extension into the trachea following blunt trauma, which was successfully managed with ECMO and surgical repair.

## CASE PRESENTATION

**Fig. 1:**
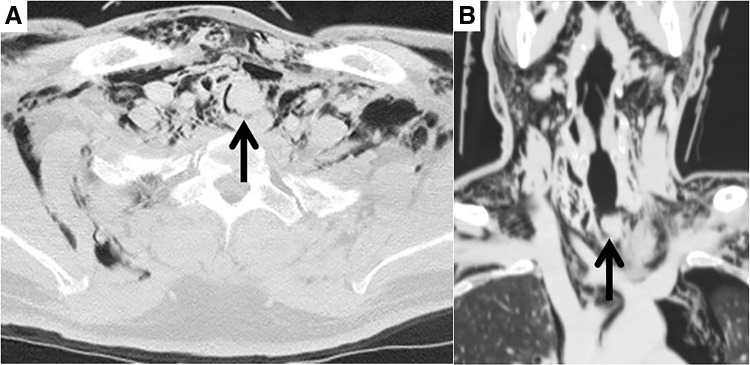
Chest computed tomography scan showing severe subcutaneous and mediastinal emphysema and tracheal injury with a 13-mm endotracheal mass (arrow). (**A**) Horizontal section. (**B**) Coronal section.

**Fig. 2:**
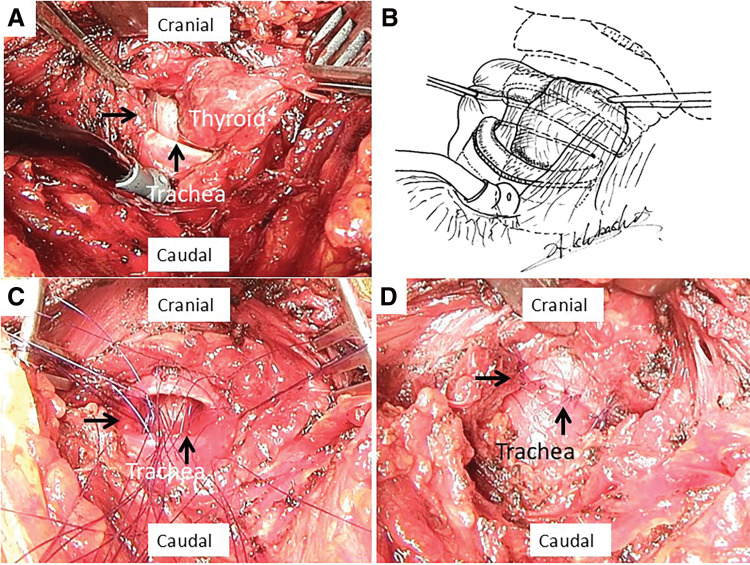
Intraoperative findings. (**A**) The inferior thyroid gland extends into the trachea from the site of tracheal injury. (**B**) Schema of **Fig. 2A** shows the thyroid gland extending into the trachea from the injury site. (**C**) Tracheal anastomosis was performed after trimming of blunt tracheal injury. (**D**) The trachea was sutured and anastomosed end-to-end with interrupted sutures. Arrows, tracheal transection

A 50-year-old male fell due to a convulsive seizure with loss of consciousness after lunch. He was found collapsed beside a table at home by his wife. After regaining consciousness, he complained of severe breathing difficulties and was transported to Institute of Science Tokyo Hospital at 13:20. On arrival, he was in respiratory failure with an SpO_2_ of 92%, blood pressure of 110/65 mmHg and pulse of 90 beats/min using a 10 L/min reservoir mask, with severe subcutaneous emphysema of the neck and stridor in the upper airway. Chest computed tomography revealed severe subcutaneous emphysema with mediastinal emphysema and a 13-mm endotracheal mass perforating and obstructing the trachea (**Figs. 1A** and **1B**). Flexible bronchoscopy revealed a suspected endotracheal mass obstructing the trachea. The patient’s respiratory distress markedly worsened with an SpO_2_ of 86%, blood pressure of 80/50 mmHg and pulse of 130 beats/min while using a 10 L/min reservoir mask at 14:00. Endotracheal intubation using flexible bronchoscopy was unsuccessful because of bleeding and tracheal obstruction. Emergency tracheostomy was considered risky because the distal trachea was located at the suprasternal margin of the sternum on computed tomography. Venovenous ECMO was administered at 14:15, and his general condition stabilized after extracorporeal circulation was established. Surgery was performed for the endotracheal mass perforation and tracheal obstruction. After a transverse neck incision, the peritracheal area was dissected to confirm the site of tracheal injury. The inferior part of the thyroid gland extended into the tracheal lumen through the site of the tracheal transection (**Figs. 2A** and **2B**). Intraoperative pathological examination of the endotracheal mass revealed a normal thyroid gland without any malignancy. Tracheal anastomosis was performed after trimming of blunt tracheal injury site. The trachea was sutured in an end-to-end anastomosis using interrupted 4-0 PDS-II (Ethicon, Somerville, NJ, USA) sutures (**Figs. 2C** and **2D**). Postoperative neck fixation was not implemented and the endotracheal tube was removed after confirming recurrent nerve function. The patient was discharged on postoperative day 10 without complications and has been followed as an outpatient for 3 years.

## DISCUSSION

This case report presents a critical case of tracheal obstruction caused by the thyroid gland, which extends into the trachea through a tracheal cartilage laceration. Thyroid gland hemorrhage after blunt trauma causing tracheal stenosis has been reported^2)^; however, there have been no reports of cases in which the thyroid gland extended into the injured area of the trachea, resulting in tracheal obstruction.

Various mechanisms that cause tracheal injuries due to blunt trauma have been reported.^3)^ Direct compression of the exposed anterior aspect of the neck may crush the trachea, particularly at the cricoid ring, against the posterior vertebral column, and transect the trachea. Cervical tracheal obstruction is caused by the destruction of tracheal cartilage and hemorrhage, and factors such as tracheal disruption due to blunt trauma, which present as subcutaneous emphysema, mediastinal emphysema, pneumothorax, respiratory distress, hemoptysis, and stridor. In this case, it is considered that cervical tracheal injury occurred due to a strong compression on the anterior neck, causing the lower left pole of the thyroid gland to prolapse into the tracheal lumen.

Airway assessment and management should be prioritized during a trauma survey.^4)^ While maintaining airway patency is crucial, impulsive endotracheal intubation under inappropriate circumstances should be avoided because it may further damage the injured trachea and lead to complete disruption of the tenuous airway. Blind intubation has failed to achieve an adequate airway in 76% of reported cases.^5)^ The use of a bronchoscope to guide the endotracheal tube is recommended. However, this does not imply that every case can be secured using these methods. In the present case, thoracic surgeons could not guide the endotracheal tube into the distal trachea. Furthermore, emergency tracheostomy was considered risky because the distal trachea was located at the suprasternal margin of the sternum on computed tomography. The patient's respiratory distress markedly worsened with an SpO_2_ of 86% with a 10 L/min reservoir mask, and venovenous ECMO was administered to stabilize further oxygenation maintenance.

Early surgical repair is preferred for cervical tracheal transection.^6)^ Recurrent laryngeal nerve injury can be expected in approximately 60% of patients with complete transection of the cervical trachea,^7)^ and tracheostomy is needed in case of bilateral vocal cord paralysis. In this case, the cervical tracheal injury could be safely repaired by performing respiratory management using ECMO, and intraoperative findings showed no recurrent laryngeal nerve injury, and no recurrent laryngeal nerve paralysis was observed when the endotracheal tube was removed.

## CONCLUSION

This case report presented a rare case of tracheal obstruction due to thyroid gland extension into the trachea caused by blunt tracheal injury, which was successfully treated by the rapid introduction of venovenous ECMO and surgical repair.

## ACKNOWLEDGMENTS

We would like to thank Editage (www.editage.jp) for editing the English language.

## DECLARATIONS

### Funding

No funding was received for this project.

### Authors’ contributions

Hironori Ishibashi: data curation, writing—original draft preparation, visualization.

Michi Aoki: review.

Shunichi Baba: review.

Akihiro Fujita: review.

Kenichi Okubo: supervision; writing review.

All authors have read and approved the final manuscript.

### Availability of data and materials

The data supporting the conclusions of this article are included within the article.

### Ethical approval and consent to participate

The patient provided written consent for publishing. This study was approved by the Institutional Review Board of Institute of Science Tokyo Hospital (certificate number M2022-126).

### Consent for publication

The patient provided consent for the use of his personal data.

### Competing interests

The authors declare that they have no competing interests.
